# Towards stereochemical control: A short formal enantioselective total synthesis of pumiliotoxins 251D and 237A

**DOI:** 10.3762/bjoc.9.271

**Published:** 2013-11-05

**Authors:** Jie Zhang, Hong-Kui Zhang, Pei-Qiang Huang

**Affiliations:** 1 Department of Chemistry and Fujian Provincial Key Laboratory of Chemical Biology, College of Chemistry and Chemical Engineering, Xiamen University, Xiamen, Fujian 361005, P. R. China; 2 State Key Laboratory of Applied Organic Chemistry Lanzhou University, Lanzhou 730000, P. R. China

**Keywords:** enantioselective synthesis, Grignard reagent, pumiliotoxin 237A, pumiliotoxin 251D, reductive dehydroxylation, ring closure, *trans*-methylation

## Abstract

A concise enantioselective synthesis of the advanced intermediate **5** for the synthesis of pumiliotoxins (Gallagher’s intermediate) is described. The synthesis started from the regio- and *trans*-diastereoselective (dr = 98:2) reductive 3-butenylation of (*R*)-3-(*tert*-butyldimethylsilyloxy)glutarimide **14**. After *O*-desilylation and Dess–Martin oxidation, the resulting keto-lactam **10** was subjected to a highly *trans*-stereoselective addition of the methylmagnesium iodide to give carbinol **11** as sole diastereomer. An efficient ring closure procedure consisting of ozonolysis and reductive dehydroxylation provided the indolizidine derivative **5**, which completed the formal enantioselective total synthesis of pumiliotoxins 251D and 237A.

## Introduction

**Figure 1:**
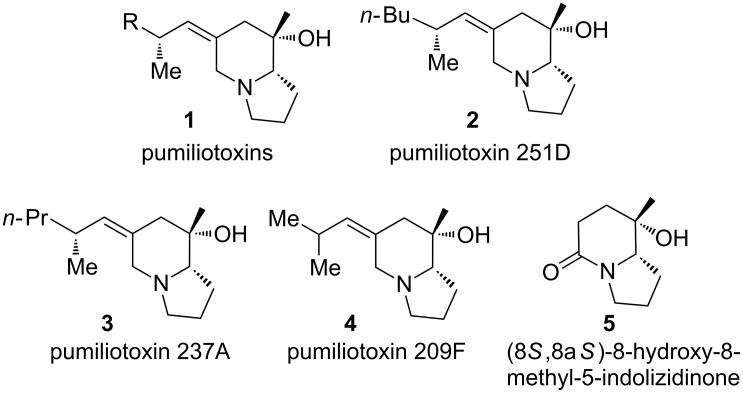
Structures of some pumiliotoxins and an advanced intermediate.

Pumiliotoxins (PTXs, **1**, Figure 1) such as pumiliotoxin 251D (**2**) are a subclass of indolizidine alkaloids isolated from the skin secretion of neotropical frogs. A total of 19 members have been isolated and partially characterized [1]. Pumiliotoxins are structurally characterized by a (*Z*)-6-alkylidene-8-hydroxy-8-methylindolizidine ring system, which distinguishes from one to another by the 6-alkylidene side chain [1]. Interestingly, it is known that poison frogs don’t produce the alkaloids themselves, instead, they accumulate alkaloids from dietary alkaloid-containing arthropods serving as a chemical defense against predators. It is not surprising that the alkaloids found in poison frogs can also be detected from ants, and most of them show remarkable bioactivities [2–3]. For example, formicine ants have been shown to be an arthropod source of the pumiliotoxin alkaloids of dendrobatid poison frogs [4–5]. Forthermore, from the extracts of an unidentified *Scheloribates sp*. of mites, pumiliotoxin 237A (**3**) was detected as a minor component [6].

That the wide range of biological activities possessed by these molecules have attracted much attention of synthetic organic chemists, and numerous approaches have been reported [7–9]. Pumiliotoxin 251D (PTX 251D) (**2**) is the first structurally defined member of pumiliotoxins, a class of dendrobatid alkaloids isolated from Ecuadorean poisonous frog, *Dendrobares tricolor* in 1980 [10]. Since the pioneering work of Overman [11], a number of approaches have been developed for the synthesis of PTX 251D [12–26]. Among them, that of Gallagher [12] has attracted much attention. They demonstrated that (8*S*,8a*S*)-8-hydroxy-8-methyl-5-indolizidinone (**5**, Figure 1) can serve as an advanced intermediate for the synthesis of pumiliotoxin 251D [12]. Later on, Nubbemeyer and co-workers used the Horner olefination to convert **5** and its diastereomer into (+)-PTX 251D (**2**) and the 8-epimer of PTX 209F (**4**), respectively [17]. Recently, (8*S*,8a*S*)-**5** has been used for the synthesis of PTX 237A (**3**) [6]. Thus, it is logic to envision that an efficient synthesis of this key intermediate would allow access to other member of pumiliotoxins and their analogues. To date more than ten synthetic approaches to (8*S*,8a*S*)-**5** have been published [12–15,18–22,27–31], among them L-proline and its derivative were used as popular precursors from the pool of chiral compounds [13–15,18–22]. Herein, we report a concise diastereoselective synthesis of (8*S*,8a*S*)-**5** starting from (*R*)-3-(*tert*-butyldimethylsilyloxy)glutarimide **14**, a versatile building block developed from our laboratory [32].

## Results and Discussion

**Scheme 1:**
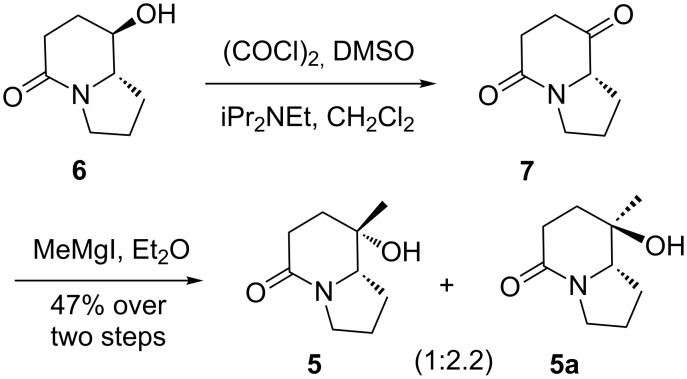
Synthesis of **5** from **6** via oxidation–addition sequence.

In recent years, we have been engaged in the development of efficient methodologies for the synthesis of nitrogen-containing bioactive heterocycles [33–37], and recently reported an approach for the synthesis of (8*R*,8a*S*)-8-hydroxy-5-indolizidinone (**6**) [38]. As a continuation of that study, the synthesis of (8*S*,8a*S*)-**5** starting from compound **6** was envisioned. For this purpose, alcohol **6** was subjected to Swern oxidation (DMSO, (COCl)_2_, iPr_2_NEt, −78 to 0 °C, 3 h), and the resulting keto-lactam **7** treated with MeMgI in diethyl ether (−78 to 0 °C, 1 h), which gave alcohols **5**/**5a** as an inseparable mixture of diastereomers in a ratio of 1:2.2 (combined yield: 47% over 2 steps) (Scheme 1). A comparison of spectral data (^1^H and ^13^C NMR) of our products **5**/**5a** with those reported [12,22,28] showed that the desired (8*S*,8a*S*)-**5** was the minor diastereomer. Similar results have been reported by Nubbemeyer [27] and Li [8]. In view of the fruitless efforts of Nubbemeyer [27] and Li [8] in inversing the diastereoselectivity of the methylation reaction of keto-lactam **7**, an alternative approach was envisaged.

**Scheme 2:**
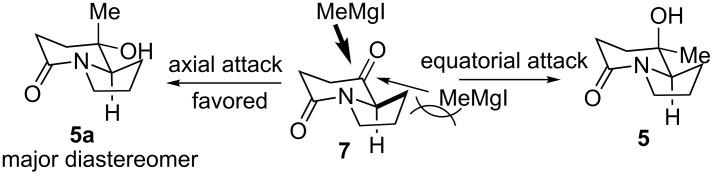
Plausible stereochemical course of the preferential axial addition of methylmagnesium iodide to bicyclic keto-lactam **7**.

To develop a diastereoselective approach to (8*S*,8a*S*)-**5**, it would be helpful to analyze the plausible stereochemical course of the methylation of keto-lactam **7**. The observed unusual stereoselective *cis*-methylation implicates a preferential axial attack of the methylmagnesium iodide to the bicyclic keto-lactam **7** (Scheme 2). Although the preferential axial attack of “small” nucleophiles to cyclohexanone is well known [39–41], the “small” nucleophiles are limited to some metallo-hydrides, metallo-acetylenide [42] and (cyanomethyl)lithium [43]. In the case of bicyclic keto-lactam **7**, presumably, the equatorial attack of methylmagnesium iodide to give **5**, suffers from unfavorable eclipsing interactions between the incoming methylmagnesium iodide and the vicinal methylene group. As a consequence, the axial attack to give **5a** is more favorable.

**Scheme 3:**
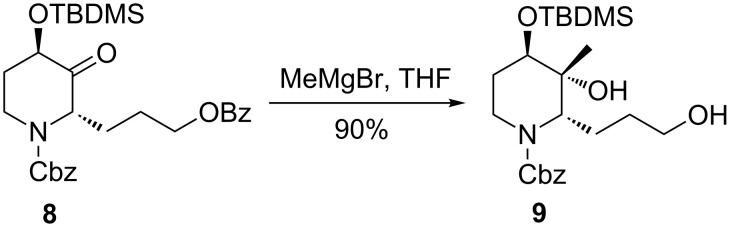
Holmes’ exclusive *trans*-diastereoselective methylation of *N*-Cbz-protected piperidin-3-one **8**.

It was envisioned that if a monocyclic keto-lactam was used, the situation would be changed and an equatorial attack of the nucleophile giving the *cis*-product would be preferable. Indeed, Holmes and co-workers have reported that methylmagnesium bromide addition to *N*-Cbz-protected piperidin-3-one **8** produced exclusively the *trans*-methylation product **9** [44] (Scheme 3). In that case, the *N*-benzyloxycarbonyl group imposed A^1,3^-strain on piperidine derivatives founded the basis for the observed stereocontrol.

**Scheme 4:**
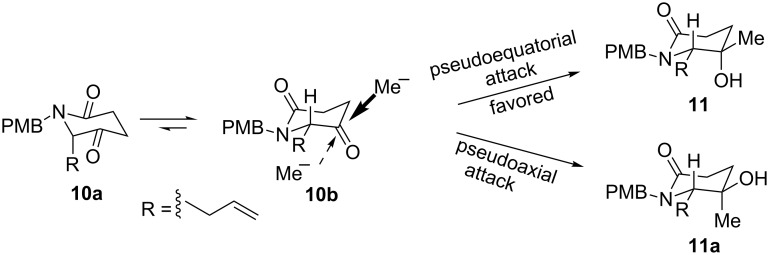
Our plan for the *trans*-diastereoselective methylation of keto-lactam **10**.

Thus, we chose keto-lactam **10** as our substrate. Although compound **10** is not a urethane, and a A^1,3^-strain not longer exists, a simple chair conformational-controlled preferential equatorial attack could be expected (Scheme 4). Substrate **10** may adopt two plausible chair conformations **10a** and **10b**, in which conformation **10b** with a pseudoequatorially positioned *N*-α-substitutent is more stable. Thus, the pseudoequatorial attack of MeMgI would be preferential, which gives the product **11** as the major product.

**Scheme 5:**
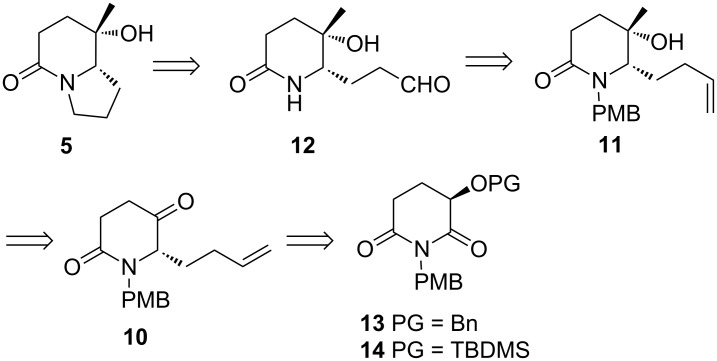
Retrosynthetic analysis of (8*S*,8a*S*)-8-hydroxy-8-methylindolizidin-5-one (**5**).

On the basis of the abovementioned analysis, a retrosynthetic analysis of (8*S*,8a*S*)-**5** is displayed in Scheme 5, which features the formation of the fused pyrrolidine ring from the but-3-ene-1-yl group, the expected *trans*-diastereoselective methylation as the key step, and protected (*R*)-3-hydroxyglutarimide-based regio- and *trans*-diastereoselective reductive alkylation, a synthetic methodology developed from our laboratory [32,38,45–46], as the starting point.

**Scheme 6:**
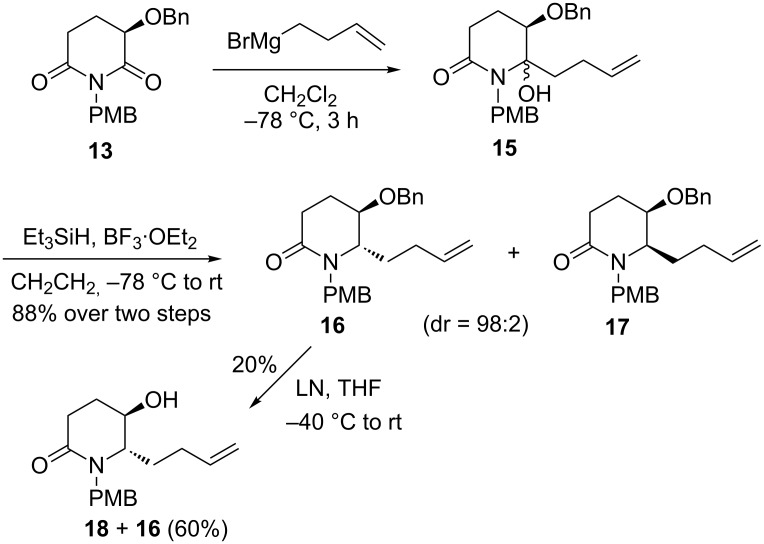
Synthesis of compound **18**.

Our synthesis started from the addition of a freshly prepared 3-butenylmagnesium bromide to imide **13** [47] in dichloromethane at −78 °C for 3 h, which gave regioselective carbinol **15** as a mixture of two diastereomers. Without separation, the mixture was treated with Et_3_SiH/BF_3_·Et_2_O (−78 °C to rt, 2 h) [32] to yield the reductive dehydroxylation products **16** and **17** in a ratio of 98:2 (determined by ^1^H NMR) [32] (combined yield: 88% over 2 steps), from which only the major product **16** was isolated (Scheme 6). For the chemoselective debenzylation, lithium naphthalenide (LN) was used [48]. To our disappointment, attempted cleavage of the benzyl group by LN (THF, −40 °C to rt, 2 h) gave the desired **18** in only 20% yield along with 60% of the recovered starting material **16**.

**Scheme 7:**
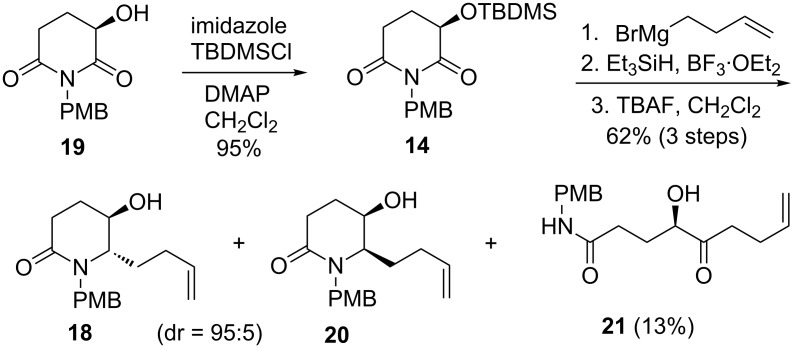
Synthesis of hydroxylactam **18**.

Due to the low yield in the debenzylation process, we decided to change the *O*-protecting group from benzyl to TBDMS, namely, use of TBDMS ether **14** as the starting material. Thus, (*R*)-3-(*tert*-butyldimethylsilyloxy)glutarimide **14** [49] was prepared from the known (*R*)-3-hydroxyglutarimide **19** (prepared from (*R*)-glutamic acid in 69% overall yield over 4 steps [47]) in a yield of 95%. Successive treatment of imide **14** with 3-butenylmagnesium bromide (CH_2_Cl_2_, −20 °C, 3 h) and the resulting hemi-aminal with Et_3_SiH/BF_3_·Et_2_O (CH_2_Cl_2_, −78 °C, 2 h, then −20 °C, 2 h) gave a diastereomeric mixture of lactams. Without separation, the mixture was treated with TBAF in dichloromethane to produce alcohols **18** and **20** (combined yield: 62% over 3 steps) in a ratio of 95:5 (determined by ^1^H NMR), along with the keto-amide **21** in 13% yield (Scheme 7). Separation of the mixture by column chromatography gave pure diastereomer **18** in 59% overall yield from **14**. The *trans*-stereochemistry was tentatively assigned to compound **18** on the basis of our previous results, which was confirmed by the conversion of **18** into the known compound **5** (vide infra).

**Scheme 8:**
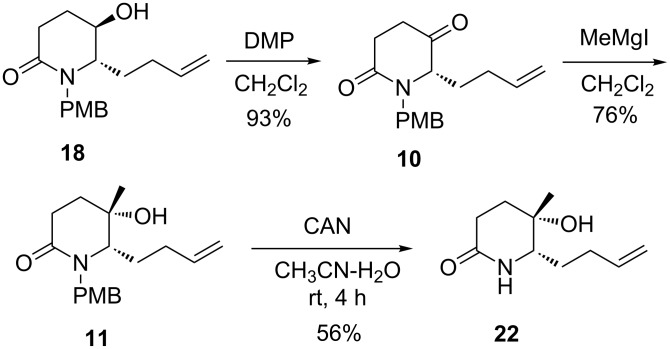
Synthesis of tertiary alcohol **22**.

With lactam **18** in hand, its conversion to novel tertiary alcohol **22** was investigated. Oxidation of alcohol **18** with an excess of Dess–Martin periodinane (DMP) in dichloromethane at room temperature for 2 h proceeded smoothly to give ketone **10** in 93% yield. It is noteworthy that keto-lactam **10** is configurational labile, and should be used immediately in the next reaction. Treatment of keto-lactam **10** with MeMgI (3 equiv) in dichloromethane (0 °C to rt, 12 h) gave a 76% yield of the desired methylation product **11** as the sole observable diastereomer (Scheme 8), whose stereochemistry was determined after converting into the known (8*S*,8a*S*)-**5**. This result verifies the previous assumption outlined in Scheme 4.

To remove the PMB protecting group, compound **11** was treated with ammonium cerium nitrate (CAN) in CH_3_CN/H_2_O (v/v 3:1) [50] at room temperature for 4 h to afford the deprotected lactam **22** in 56% yield.

**Scheme 9:**
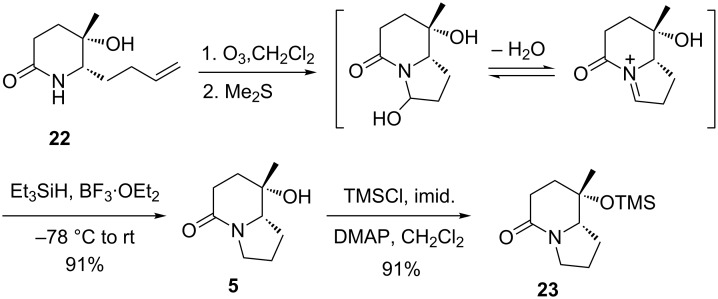
Synthesis of (8*S*,8a*S*)-**5** and its silyl ether **23**.

Our next task was the oxidative cyclization of the olefin **22** to form the indolizidinone ring. Thus, ozonolysis of olefin **22** in dichloromethane [51], followed by quenching with Me_2_S furnished the hemiaminal tautomer via intermediacy of lactam-aldehyde. Without isolation, the crude was subjected to the reductive dehydroxylation with Et_3_SiH/BF_3_·Et_2_O (CH_2_Cl_2_, −78 °C) to give the desired indolizidinone **5** in an overall yield of 91% from **22** (Scheme 9).

The spectral data of the our synthetic (8*S*,8a*S*)-**5** are in agreement with those reported [12,22,27–29]. Due to the differences between the reported optical rotation values {[*α*]_D_^20^ −42.7 (*c* 1.0, CHCl_3_); lit. [12] [*α*]_D_^21^ −47.0 (*c* 0.97, CHCl_3_); lit. [22] [*α*]_D_^22^ −55 (*c* 0.79, CHCl_3_); lit. [28] [*α*]_D_^25^ −41.3 (*c* 0.48, CHCl_3_); lit. [29] [*α*]_D_^25^ −32.1 (*c* 1.0, CHCl_3_)}, compound **5** was converted into the known silyl ether **23** (TMSCl, imidazole, DMAP, CH_2_Cl_2_, 0 °C to rt, 24 h, 91% yield), which is identical in all aspects with that reported by Nubbemeyer {[*α*]_D_^20^ −35.3 (*c* 0.89, CHCl_3_); lit. [27] [*α*]_D_^20^ −35.1 (*c* 1.08, CHCl_3_)}. Silyl ether **23** may serve as an advanced intermediate in the synthesis of pumiliotoxin 209F [17].

## Conclusion

In summary, an eight-step synthesis of the advanced intermediate (8*S*,8a*S*)-**5** for the syntheis of pumiliotoxins has been achieved in 21% overall yield starting from (*R*)-3-silyloxyglutarimide derivative **14**. The method is based on the versatile building block **14** and relied on a highly diastereoselective *trans*-addition of methylmagnesium iodide to keto-lactam **10**. Since compound (8*S*,8a*S*)-**5** has been converted into pumiliotoxins 251D (**2**) and 237A (**3**), by Gallagher, Nubbemeyer, and Mori, respectively, its synthesis constitutes a formal enantioselective total synthesis of pumiliotoxins 251D and 237A. Compound (8*S*,8a*S*)-**5** would be of value for the synthesis of other pumiliotoxins as well. The highly diastereoselective *trans*-addition of a methylmagnesium iodide to keto-lactam **10** provides a new example of achieving the desired diastereoselection simply by the chair-conformation control [52–53].

## Experimental

Optical rotations were recorded on a Perkin-Elmer 341 automatic polarimeter. ^1^H NMR and ^13^C NMR spectra were recorded on bruker 400 spectrometer. ^1^H NMR spectra were measured in CDCl_3_, and chemical shifts are expressed in parts per million (ppm) relative to internal Me_4_Si. IR spectra were recorded on a Nicolet Avatar 330 RT-IR spectrophotometer. Mass spectra were recorded by Bruker Dalton Esquire 3000 plus and Finnigan Mat-LCQ (ESI direct injection). HRFABMS spectra were recorded on a Bruker APEX-FTMS apparatus. Elemental analyses were performed using a Vario RL analyzer. Melting points were determined on a Yanaco MP-500 melting point apparatus and are uncorrected.

Tetrahydrofuran (THF) was distilled prior to use from sodium benzophenone ketyl. Dichloromethane was distilled from phosphorus pentoxide. Silica gel (zhifu, 300–400 mesh) from Yantai silica gel factory (China) was used for column chromatography, eluting (unless otherwise stated) with ethyl acetate/petroleum ether (PE) (60–90 °C) mixture.

**(*R*)-3-(*tert*-Butyldimethylsilyloxy)-1-(4-methoxybenzyl)piperidine-2,6-dione (14):** To a solution of (*R*)-3-hydroxy-1-(4-methoxybenzyl)piperidine-2,6-dione **19** [47] (125 mg, 0.5 mmol), DMAP (10 mg) and imidazole (67 mg, 1 mmol) in CH_2_Cl_2_ (15 mL) was added TBDMSCl (52 μL, 0.6 mmol). The mixture was stirred at room temperature for 24 h before quenching with H_2_O (5 mL). The organic layer was separated and the aqueous phase extracted with CH_2_Cl_2_ (10 mL × 5). The combined organic layers were washed with brine, dried over anhydrous Na_2_SO_4_, filtered and concentrated under reduced pressure. The residue was purified by column chromatography on silica gel (EtOAc/hexane 1:2) to give silyl ether **14** (517 mg, yield: 95%) as a colorless oil. [*α*]_D_^20^ +19.2 (*c* 1.1, CHCl_3_); IR (film) ν_max_: 3062, 2936, 1728, 1658, 1494, 1451, 1367 cm^−1^; ^1^H NMR (400 MHz, CDCl_3_) δ 0.11 (s, 3H), 0.12 (s, 3H), 0.87 (s, 9H), 1.92–2.07 (m, 2H), 2.59 (ddd, *J* = 17.7, 7.5, 5.4 Hz, 1H), 2.91 (ddd, *J* = 17.7, 8.1, 5.4 Hz, 1H), 3.76 (s, 3H), 4.30 (dd, *J* = 7.4, 4.0 Hz, 1H), 4.83 (d, *J* = 14.0 Hz, 1H), 4.86 (d, *J* = 14.0 Hz, 1H), 6.79 (d, *J* = 8.7 Hz, 2H), 7.30 (d, *J* = 8.7 Hz, 2H); ^13^C NMR (100 MHz, CDCl_3_) δ −5.4, −4.7, 18.2, 25.6 (3C), 26.4, 29.1, 42.4, 55.2, 69.3, 113.7 (2C), 129.4, 130.3 (2C), 158.9, 171.8, 172.2; HRMS ESI (*m*/*z*): [M + Na]^+^ calcd for C_19_H_29_NO_4_SiNa, 386.1758; found, 386.1757.

**(5*R*,6*S*)-6-(But-3-enyl)-5-hydroxy-1-(4-methoxybenzyl)piperidin-2-one (18):** To a solution of compound **14** (2.8 g, 7.71 mmol) in anhydrous CH_2_Cl_2_ (100 mL) at −20 °C was added dropwise a freshly prepared 3-butenylmagnesium bromide (1 M in THF, 15 mL, 15 mmol). After being stirred at −20 °C for 3 h, the reaction was quenched with a saturated aqueous solution of NH_4_Cl (15 mL). The organic layer was separated and the aqueous phase was extracted with CH_2_Cl_2_ (25 mL × 5). The combined organic layers were washed with brine (10 mL × 5), dried over anhydrous Na_2_SO_4_, filtered and concentrated under reduced pressure. Without further separation, the crude product (2.4 g, 5.7 mmol) was treated with Et_3_SiH (9 mL, 57 mmol) and BF_3_·Et_2_O (2.12 mL, 17.2 mmol) in CH_2_Cl_2_ (100 mL) at −78 °C. The mixture was stirred at −78 °C for 2 h, and then allowed to warm to −20 °C. After being stirred for another 2 h, saturated aqueous solution of NaHCO_3_ (25 mL) was added and aqueous layer was extracted with CH_2_Cl_2_ (15 mL × 5). The combined organic layers were washed with brine, dried over anhydrous Na_2_SO_4_, filtered and concentrated under reduced pressure to give a crude product, which was used in the next step without further purification. To a solution of the crude product in THF (50 mL) was added a 1 M solution of TBAF in THF (17.1 mL, 17.1 mmol) at 0 °C. The mixture was allowed to warm to room temperature. After being stirred for 4 h at room temperature, the reaction was quenched with water, and extracted with CH_2_Cl_2_ (15 mL × 5). The combined organic phases were washed with brine, dried over anhydrous Na_2_SO_4_, filtered and concentrated under reduced pressure. The residue was purified by column chromatography on silica gel (EtOAc/hexane 2:1) to give diastereomers **18** (969 mg, yield: 59%) and **20** (51 mg, yield: 3%). Compound **18**: white solid, mp 102–105 °C (EtOAc/hexane); [α]_D_^20^ −65.0 (*c* 1.0, CHCl_3_); IR (film) ν_max_: 3375, 2934, 1612, 1512, 1193, 1056, 784, 560 cm^−1^; ^1^H NMR (400 MHz, CDCl_3_) δ 1.37–1.49 (m, 1H), 1.67–1.64 (m, 2H), 1.91–2.00 (m, 2H), 2.28 (ddd, *J* = 18.1, 7.2, 2.8 Hz, 1H), 2.58 (ddd, *J* = 18.1, 10.5, 7.6 Hz, 2H), 3.20 (dt, *J* = 9.4, 2.6 Hz, 1H), 3.72 (s, 3H), 3.81 (d, *J* = 14.9 Hz, 1H), 3.93 (dt, *J* = 4.6, 2.6 Hz, 1H), 4.91–5.01 (m, 2H), 5.14 (d, *J* = 14.9 Hz, 1H), 5.69 (ddt, *J* = 16.9, 10.3, 6.6 Hz, 1H), 6.78 (d, *J* = 8.6 Hz, 2H), 7.15 (d, *J* = 8.6 Hz, 2H); ^13^C NMR (100 MHz, CDCl_3_) δ 23.9, 26.7, 30.0, 31.3, 47.3, 55.1, 61.8, 65.0, 113.8 (2C), 115.5, 129.0, 129.1 (2C), 136.9, 158.7, 169.8; HRMS ESI (*m*/*z*): [M + Na]^+^ calcd for C_17_H_23_NO_3_Na, 312.1570; found, 312.1570.

**(*S*)-6-(But-3-en-1-yl)-1-(4-methoxybenzyl)piperidine-2,5-dione (10):** To a stirred solution of compound **18** (100 mg, 0.34 mmol) in CH_2_Cl_2_ (5 mL) was added Dess–Martin periodinane (220 mg, 0.52 mmol) at room temperature. After being stirred for 2 h, the reaction was quenched with a 10% aqueous solution of Na_2_S_2_O_3_. The aqueous phase was extracted with EtOAc (15 mL × 3). The combined organic layers were washed successively with a saturated aqueous solution of NaHCO_3_ (5 mL × 3) and brine (5 mL × 2). The combined organic phases were dried over Na_2_SO_4_, filtered and concentrated under reduced pressure. The residue was purified by column chromatography on silica gel (EtOAc/hexane 1:2) to give compound **10** (98 mg, yield: 93%) as a colorless oil. [α]_D_^20^ +37.5 (*c* 1.0, CHCl_3_); IR (film) ν_max_: 2955, 2926, 2834, 1725, 1625, 1512, 1246, 1173, 1032 cm^−1^; ^1^H NMR (400 MHz, CDCl_3_) δ 1.79–2.11 (m, 4H), 2.57–2.69 (m, 2H), 2.73–2.80 (m, 2H), 3.68 (dd, *J* = 7.5, 4.6 Hz, 1H), 3.79 (s, 3H), 3.85 (d, *J* = 14.7 Hz, 1H), 4.97–5.08 (m, 2H), 5.28 (d, *J* = 14.7 Hz, 1H), 5.70 (ddt, *J* = 16.7, 10.2, 6.4 Hz, 1H), 6.84 (d, *J* = 8.7 Hz, 2H), 7.15 (d, *J* = 8.7 Hz, 2H); ^13^C NMR (100 MHz, CDCl_3_) δ 29.0, 29.2, 30.6, 35.3, 47.2, 55.2, 63.9, 114.2 (2C), 116.3, 128.3, 129.5 (2C), 136.4, 159.3, 169.6, 206.2; HRMS ESI (*m*/*z*): [M + Na]^+^ calcd for C_17_H_21_NO_3_Na, 310.1414; found, 310.1419.

**(5*S*,6*S*)-6-(But-3-enyl)-5-hydroxy-1-(4-methoxybenzyl)-5-methylpiperidin-2-one (11):** To a solution of compound **10** (92 mg, 0.32 mmol) in anhydrous CH_2_Cl_2_ (100 mL) was added dropwise a freshly prepared 1 M diethyl ether solution of CH_3_MgI (1.0 mL, 1.0 mmol) at 0 °C. After being stirred at room temperature overnight, the reaction was quenched with a saturated aqueous solution of NH_4_Cl (5 mL). The organic layer was separated and the aqueous phase was extracted with CH_2_Cl_2_ (15 mL × 5). The combined organic layers were washed with brine, dried over anhydrous Na_2_SO_4_, filtered and concentrated under reduced pressure. The residue was purified by column chromatography on silica gel (EtOAc/hexane 1:1) to give compound **11** (75 mg, yield: 76%) as a colorless oil. [α]_D_^20^ −73.5 (*c* 1.0, CHCl_3_); IR (film) ν_max_: 3378, 2928, 2925, 2874, 1612, 1512, 1247, 1150, 1034, 914, 847 cm^−1^; ^1^H NMR (400 MHz, CDCl_3_) δ 0.99 (s, 3H), 1.57–1.65 (m, 2H), 1.73 (d, *J* = 16.5 Hz, 1H), 2.04–2.12 (m, 2H), 2.20–2.27 (m, 2H), 2.41 (ddd, *J* = 18.6, 9.5, 8.8 Hz, 1H), 2.55 (ddd, *J* = 18.6, 8.8, 2.2 Hz, 1H), 3.00 (td, *J* = 5.4, 1.6 Hz, 1H), 3.59 (d, *J* = 14.3 Hz, 1H), 3.78 (s, 3H), 5.00–5.10 (m, 2H), 5.45 (d, *J* = 14.3 Hz, 1H), 5.81 (ddt, *J* = 16.8, 10.2, 6.7 Hz, 1H), 6.83 (d, *J* = 8.6 Hz, 2H), 7.20 (d, *J* = 8.6 Hz, 2H); ^13^C NMR (100 MHz, CDCl_3_) δ 26.9, 29.0, 30.5, 30.8, 32.5, 48.6, 55.2, 63.1, 70.1, 113.8 (2C), 115.5, 129.0, 130.4 (2C), 138.0, 159.1, 168.7; HRMS ESI (*m*/*z*): [M + Na]^+^ calcd for C_18_H_25_NO_3_Na, 326.1727; found, 326.1728.

**(5*S*,6*S*)-6-(But-3-enyl)-5-hydroxy-5-methylpiperidin-2-one (22):** To a solution of compound **11** (463 mg, 1.52 mmol) in a mixture of CH_3_CN (32 mL) and H_2_O (11 mL) was added ammonium cerium nitrate (2.5 g, 4.56 mmol) in one portion. The mixture was stirred for 4 h at room temperature. To the resulting mixture was added H_2_O (5 mL), and the mixture was extracted with EtOAc (30 mL × 5). The combined organic layers were washed successively with a saturated solution aqueous of NaHCO_3_ and brine. The organic phases were dried over anhydrous Na_2_SO_4_, filtered and concentrated under reduced pressure. The residue was purified by column chromatography on silica gel (MeOH/CH_2_Cl_2_ 1:40) to give compound **22** (153 mg, yield: 56%) as a pale yellow oil. [α]_D_^20^ −43.0 (*c* 1.18, CHCl_3_); IR (film) ν_max_: 3366, 2932, 1612, 1475, 1406, 1312, 919 cm^−1^; ^1^H NMR (400 MHz, CDCl_3_) δ 1.31 (s, 3H), 1.52–1.61 (m, 1H), 1.76–1.93 (m, 3H), 2.10 (m, 1H), 2.22–2.35 (m, 2H), 2.57 (ddd, *J* = 18.2, 11.1, 7.0 Hz, 1H), 2.80 (s, 1H, OH, D_2_O exchangeable), 3.17 (dd, *J* = 10.2, 1.8 Hz, 1H), 5.03–5.12 (m, 2H), 5.79 (ddt, *J* = 17.0, 10.2, 6.6 Hz, 1H), 6.03 (s, 1H, NH); ^13^C NMR (100 MHz, CDCl_3_) δ 26.3, 27.5, 28.7, 30.0, 34.1, 60.1, 67.9, 115.9, 137.1, 172.4; HRMS ESI (*m*/*z*): [M + Na]^+^ calcd for C_10_H_17_NO_2_Na, 206.1151; found, 206.1160.

**(8*S*,8a*S*)-8-Hydroxy-8-methyloctahydroindolizidin-5-one (5):** To a stirred solution of compound **22** (90 mg, 0.49 mmol) in a mixture of CH_2_Cl_2_ (8 mL) and MeOH (2 mL) was bubbled O_3_ at −78 °C for 10 min. The reaction was quenched with Me_2_S (0.2 mL). The mixture was allowed to warm to room temperature. The organic layer was separated, and the aqueous phase was extracted with CH_2_Cl_2_ (15 mL × 5). The combined organic layers were washed with brine, dried over anhydrous Na_2_SO_4_, filtered and concentrated under reduced pressure. Without further purification, to a solution of the crude mixture in CH_2_Cl_2_ (5 mL) was added Et_3_SiH (0.77 mL, 4.9 mmol) and BF_3_·Et_2_O (0.18 mL, 1.47 mmol) at −78 °C. The mixture was allowed to warm slowly to the room temperature. A saturated aqueous solution of NaHCO_3_ (2 mL) was added and aqueous phase was extracted with CH_2_Cl_2_ (15 mL × 5). The combined organic layers were washed with brine, dried over anhydrous Na_2_SO_4_, filtered and concentrated under reduced pressure. The residue was purified by column chromatography on silica gel (MeOH/CH_2_Cl_2_ 1:20) to give compound **5** (75 mg, yield: 91%) as a colorless waxy solid. [α]_D_^20^ −42.7 (*c* 1.0, CHCl_3_) {lit. [12] [α]_D_^20^ −47.0 (*c* 0.8, CHCl_3_) lit. [28] [*α*]_D_^25^ −41.3 (*c* 0.48, CHCl_3_); lit. [29] [*α*]_D_^25^ −32.1 (*c* 1.0, CHCl_3_)}; IR (film) ν_max_: 3364, 2926, 2877, 1612, 1469, 1265, 740, 703 cm^−1^; ^1^H NMR (400 MHz, CDCl_3_) δ 1.29 (s, 3H), 1.74–1.97 (m, 6H), 2.20 (br s, 1H, OH, D_2_O exchangeable), 2.38 (dd, *J* = 18.4, 7.4 Hz, 1H), 2.53 (ddd, *J* = 18.4, 11.7, 7.4 Hz, 1H), 3.35 (dd, *J* = 10.3, 5.3 Hz, 1H), 3.48–3.53 (m, 2H); ^13^C NMR (100 MHz, CDCl_3_) δ 22.0, 26.3, 26.4, 28.0, 35.1, 45.7, 66.1, 67.7, 169.0; HRMS ESI (*m*/*z*): [M + Na]^+^ calcd for C_9_H_15_NO_2_Na, 192.0995; found, 192.0999.

**(8*S*,8a*S*)-8-Methyl-8-trimethylsilyloxyoctahydroindolizidin-5-one (23):** TMSCl (25 μL, 0.28 mmol) was added a solution of compound **5** (40 mg, 0.24 mmol), DMAP (5 mg) and imidazole (32 mg, 0.48 mmol) in CH_2_Cl_2_ (8 mL) at 0 °C. The mixture was stirred at room temperature for 24 h, and then diluted with H_2_O (2 mL). The organic layer was separated and the aqueous phase was extracted with CH_2_Cl_2_ (5 mL × 5). The combined organic layers were washed with brine, dried over anhydrous Na_2_SO_4_, filtered and concentrated under reduced pressure. The residue was purified by column chromatography on silica gel (EtOAc/hexane 1:2) to give compound **23** (53 mg, yield: 91%) as a pale yellow oil. [α]_D_^20^ −35.3 (*c* 0.89, CHCl_3_) {lit. [27] [α]_D_^20^ −35.1 (*c* 1.08, CHCl_3_)}; IR (film) ν_max_: 2955, 2880, 1621, 1470, 1413, 1378, 1316, 1273, 1265, 1253, 1224, 1134, 1068, 1021 cm^−1^; ^1^H NMR (400 MHz, CDCl_3_) δ 0.05 (s, 9H), 1.26 (s, 3H), 1.60–1.90 (m, 6H), 2.25–2.33 (m, 1H), 2.33–2.45 (m, 1H), 3.15–3.20 (dd, *J* = 10.0, 5.5 Hz, 1H), 3.38–3.48 (m, 2H); ^13^C NMR (100 MHz, CDCl_3_) δ 2.1, 21.9, 26.2, 26.3 , 28.2, 35.2, 45.7, 67.3, 70.4, 168.9; HRMS ESI (*m*/*z*): [M + Na]^+^ calcd for C_12_H_23_NO_2_SiNa , 264.1390; found, 264.1392.

## Supporting Information

File 1 ^1^H and ^13^C NMR of key compounds.
