# Supplementary material for: Towards stereochemical control: A short formal enantioselective total synthesis of pumiliotoxins 251D and 237A
Source: Beilstein J Org Chem. 2013 Nov 5;9:2358–66. doi: 10.3762/bjoc.9.271 (PMC3869269; doi:10.3762/bjoc.9.271)

Towards stereochemical control: A short formal enantioselective total synthesis of pumiliotoxins 251D and 237A

Jie Zhang[1], Hong-Kui Zhang*[1] and Pei-Qiang Huang*[1,2]

Address: [1]Department of Chemistry and Fujian Provincial Key Laboratory of Chemical Biology, College of Chemistry and Chemical Engineering, Xiamen University, Xiamen, Fujian 361005, P. R. China and [2]State Key Laboratory of Applied Organic Chemistry Lanzhou University, Lanzhou 730000, P. R. China

Email: Hong-Kui Zhang - hkzhang@xmu.edu.cn; Pei-Qiang Huang - pqhuang@xmu.edu.cn

* Corresponding author

^{1}H and ^{13}C NMR of key compounds

^{1}H and ^{13}C NMR spectra of compound **14**:

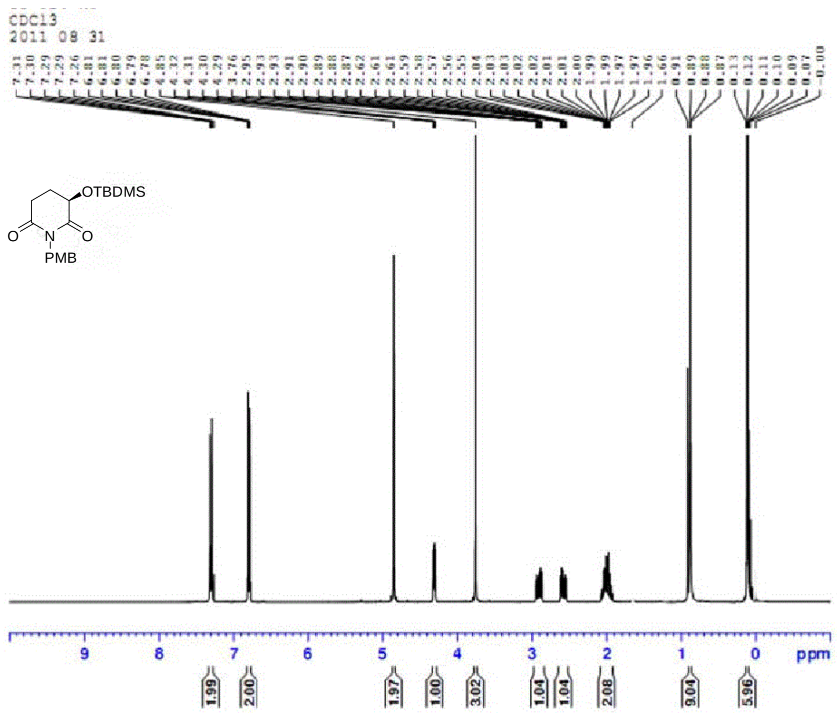

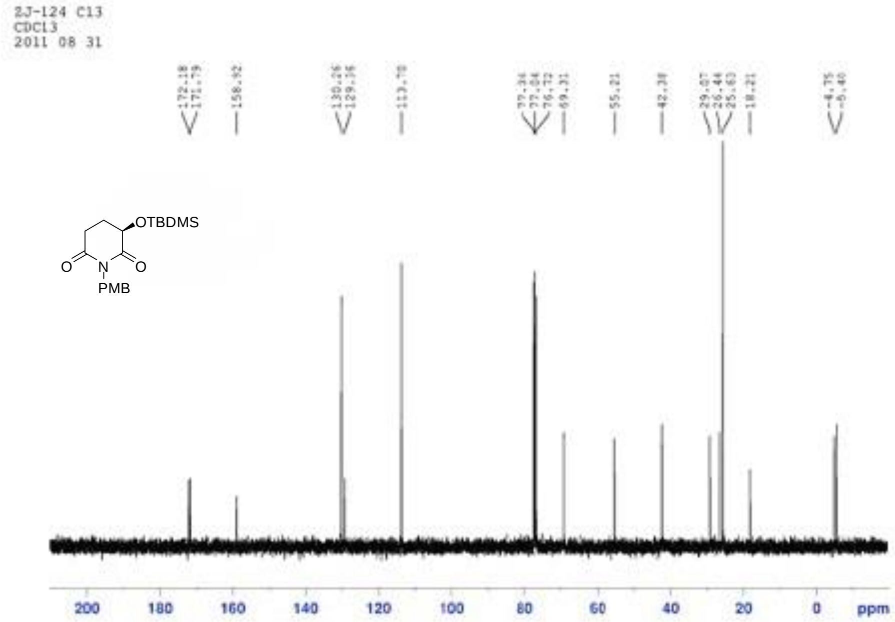

^{1}H and ^{13}C NMR spectra of compound **18**:

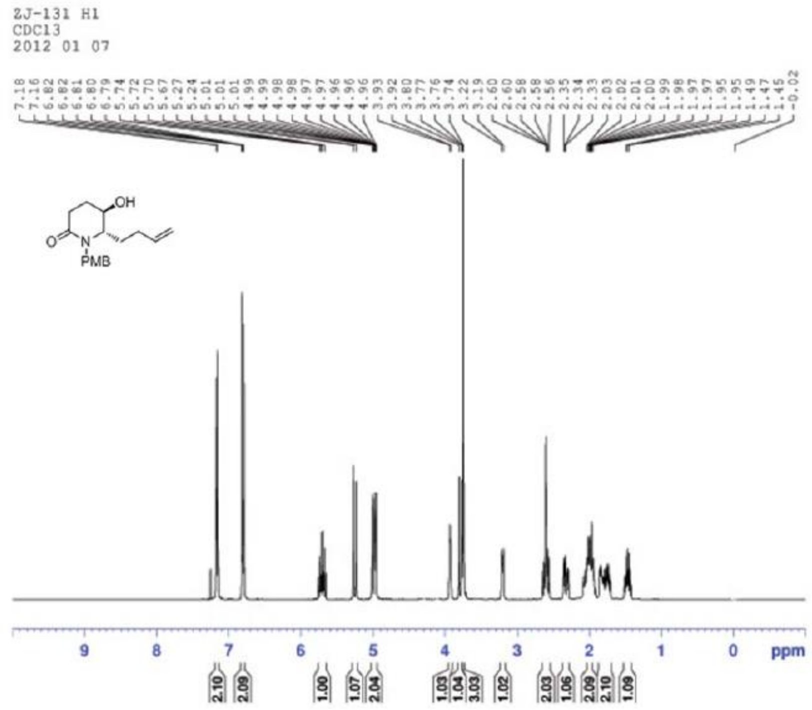

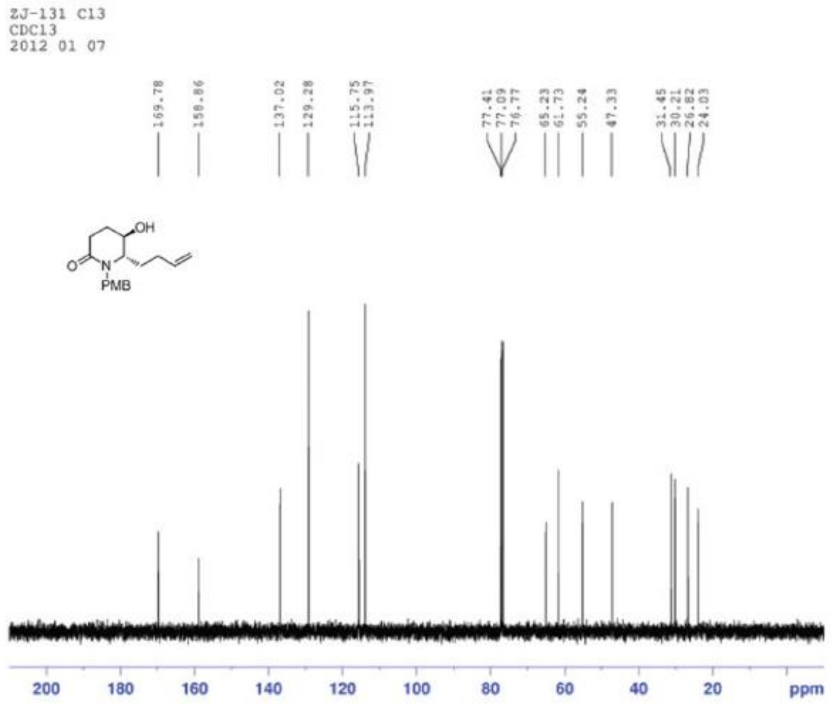

^{1}H and ^{13}C NMR spectra of compound **10**:

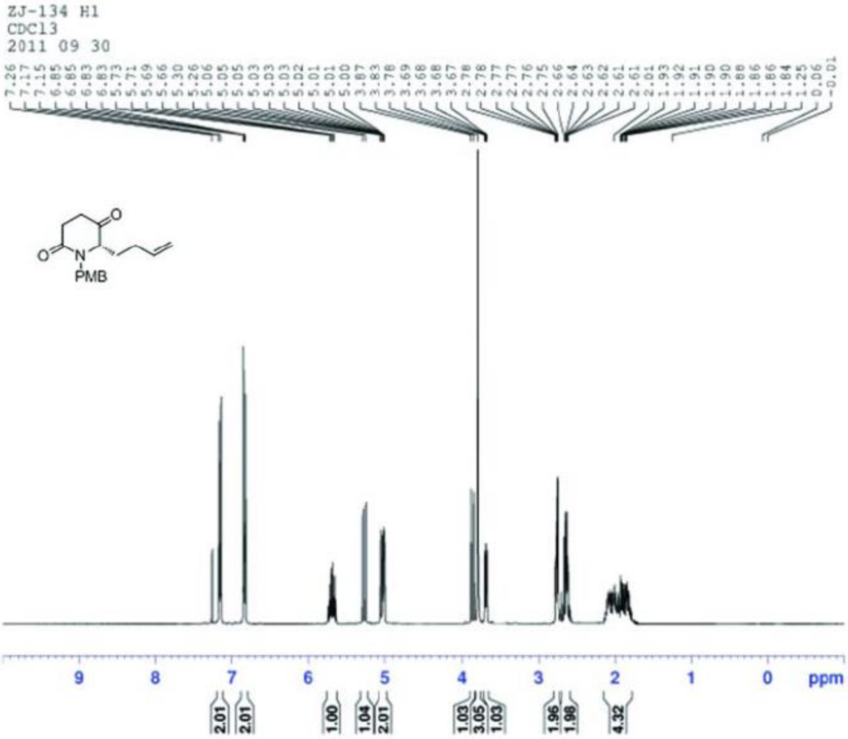

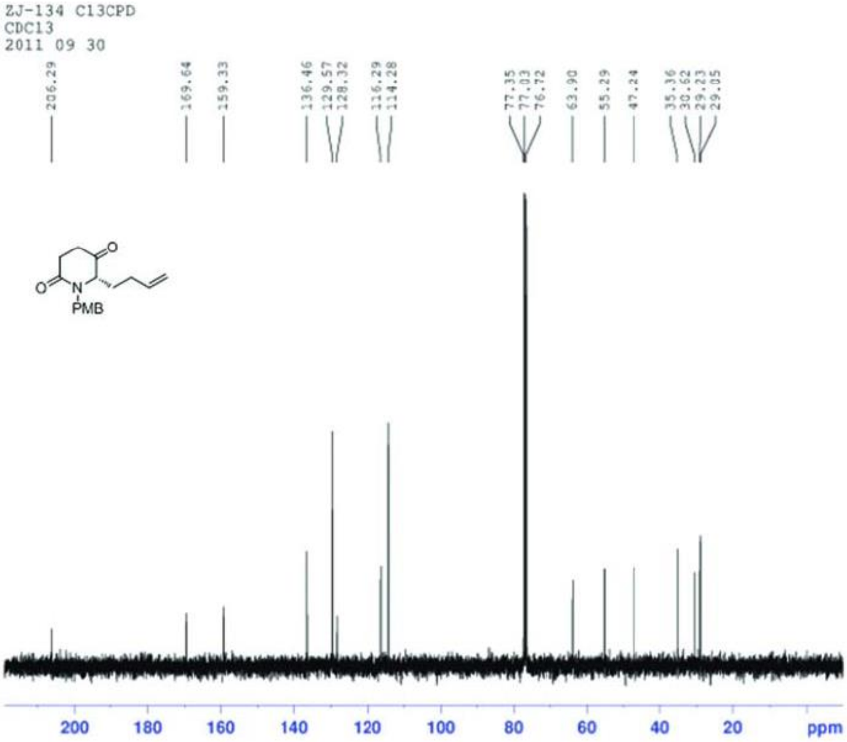

^{1}H and ^{13}C NMR spectra of compound **11**:

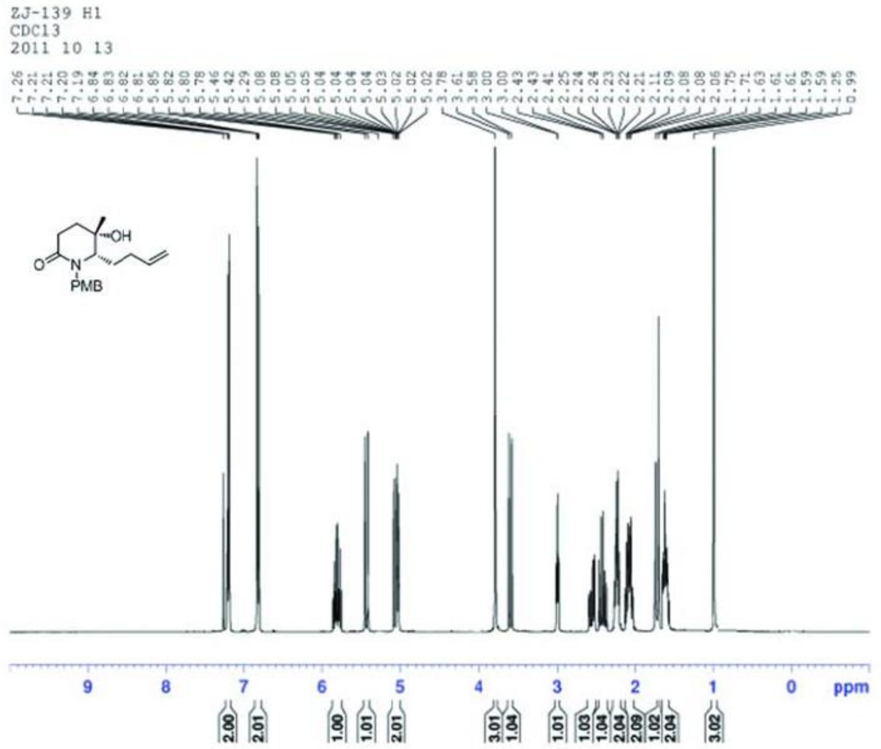

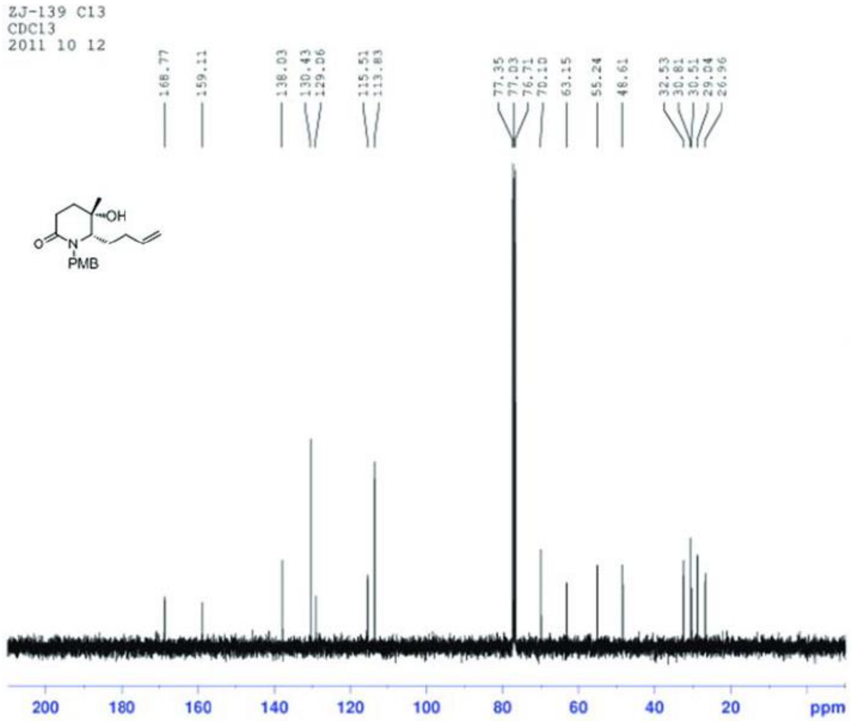

^{1}H and ^{13}C NMR spectra of compound **22**:

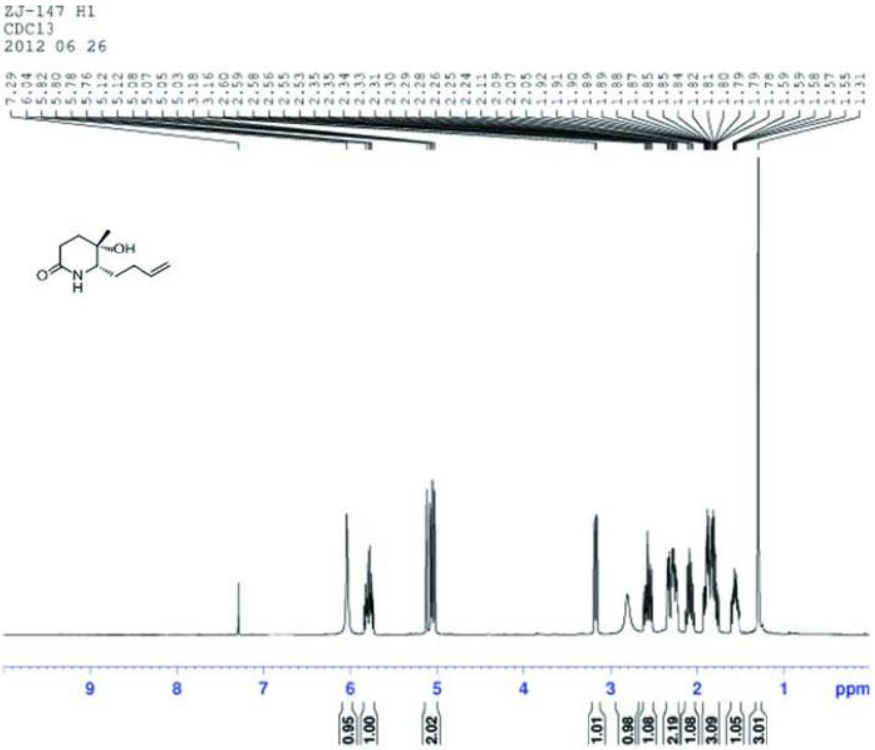

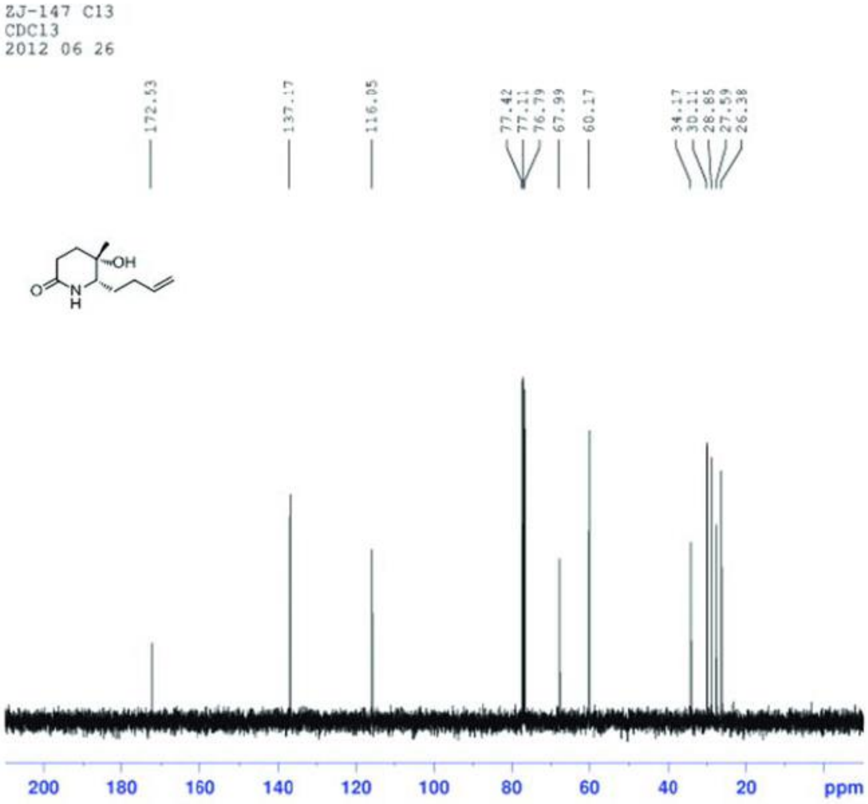

^{1}H and ^{13}C NMR spectra of compound **5**:

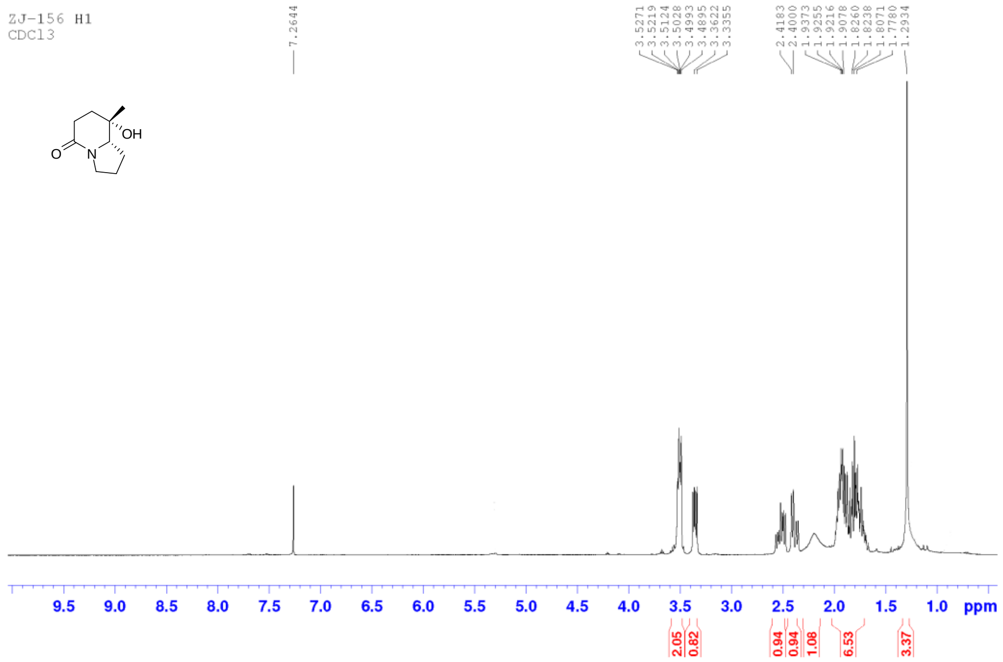

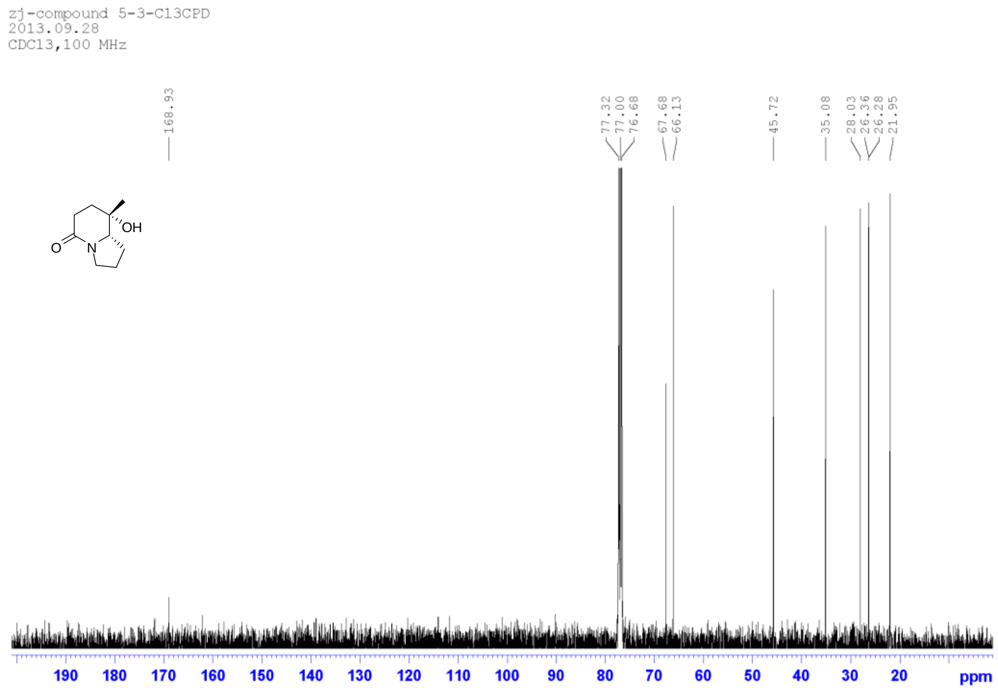

Create PDF files without this message by purchasing novaPDF printer (http://www.novapdf.com)

^{1}H and ^{13}C NMR spectra of compound **23**:

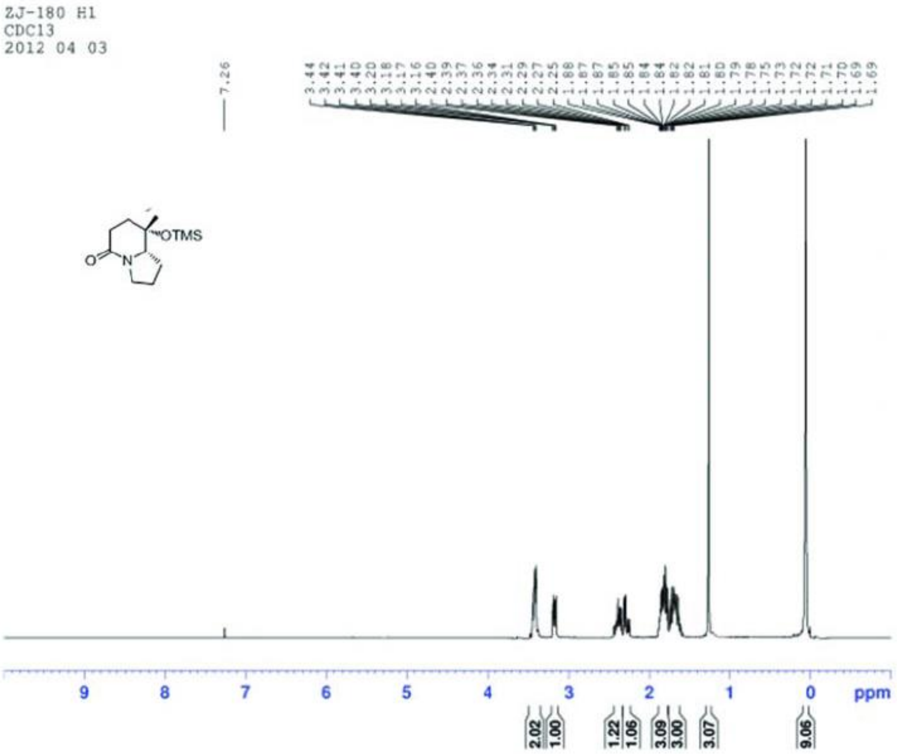

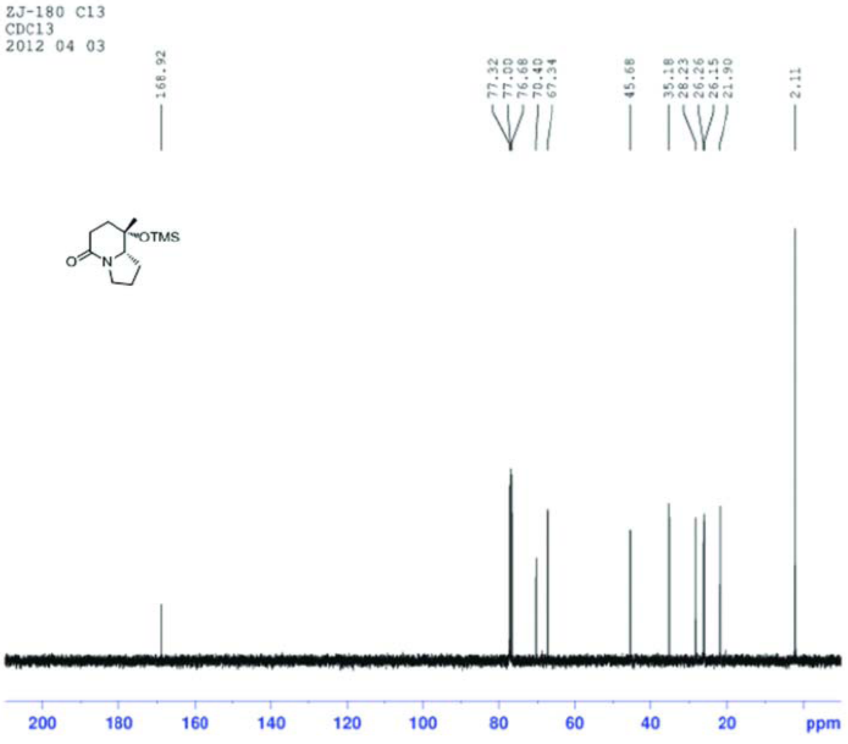

Supplement: File 1 — 1H and 13C NMR of key compounds. [file Beilstein_J_Org_Chem-09-2358-s001.pdf]
